# Renal Cell Carcinoma With Inferior Vena Cava Tumor Thrombus in Two Patients With Previous Coronary Artery Bypass Graft: Strategy for Surgical Removal

**DOI:** 10.3389/fsurg.2021.676245

**Published:** 2021-05-10

**Authors:** Gaetano Ciancio, Ahmed Farag, Tomas Salerno

**Affiliations:** ^1^Department of Surgery, Jackson Memorial Hospital, University of Miami Miller School of Medicine, Miami, FL, United States; ^2^Division of Transplantation, Jackson Memorial Hospital, University of Miami Miller School of Medicine, Miami, FL, United States; ^3^Division of Urology, Jackson Memorial Hospital, University of Miami Miller School of Medicine, Miami, FL, United States; ^4^Miami Transplant Institute, Jackson Memorial Hospital, University of Miami Miller School of Medicine, Miami, FL, United States; ^5^Department of Surgery, Zagazig University School of Medicine, Zagazig, Egypt; ^6^Division of Cardiothoracic Surgery, Jackson Memorial Hospital, University of Miami Miller School of Medicine, Miami, FL, United States

**Keywords:** renal cell carcinoma, tumor thrombus, right atrium, cardiopulmonary bypass, coronary artery bypass grafting, thrombectomy

## Abstract

Surgical management of renal cell carcinoma (RCC) with tumor thrombus (TT) extending into the inferior vena cava (IVC) and up to the hepatic veins and right atrium (RA) continues to be problematic and a challenging surgical operation. It becomes even more complicated when performing a re-sternotomy and cardiopulmonary bypass (CPB) in patients with previous coronary artery bypass grafting (CABG). Here, we report on two patients with previous CABG who presented with RCC and TT extending into the hepatic vein and above the diaphragm. These two patients underwent successful surgical resection and TT thrombectomy without the need of CBP. Recommendations are made for successfully accomplishing such surgical resections, including adequate prior preparation for the possible need to perform re-sternotomy and CPB with a coordinated team effort.

## Introduction

Renal cell carcinoma (RCC) infrequently extends into the renal vein and inferior vena cava (IVC) (1, 2), and surgical removal is the mainstay treatment of this complex tumor (3, 4). To our knowledge, there are no surgical approaches or techniques described for surgical management without using cardiopulmonary bypass (CPB) of large renal masses with tumor thrombus (TT) inferior and superior to the diaphragm in patients with a previous coronary artery bypass graft (CABG).

We herein report two patients with RCC, one with TT inferior to the diaphragm, and the other with TT superior to the diaphragm but inferior to the right atrium. Both patients had prior CABG, for 2 and 3 vessel coronary artery disease (CAD), respectively. Redo sternotomy due to prior CABG is technically difficult to perform, as adhesions and risk of injury to critical structures lying underneath the sternum, e.g., grafts, right ventricle and others clearly exist (5). Here, in both patients, the TT was removed trans-abdominally, avoiding sternotomy and CPB. The emphasis of this manuscript is to outline a strategy for dealing with such tumors without having to perform CPB (and thus, a re-sternotomy). In addition, we plan to outline a strategy for having adequate preparation should CPB become necessary electively or emergently.

## Report of Two Cases

Two male patients, Patient #1 and Patient #2, were 56 and 68 years-old, respectively, presented to outside hospitals with right-sided abdominal pain. Computed tomography of chest and abdomen, magnetic resonance imaging and echocardiogram revealed a large right renal mass with a largest diameter of 5 cm in Patient #1 and 6.5 cm in Patient #2. Both masses extended into the IVC but inferior to the right atrium (RA). Both patients had prior CABG for 2 and 3 vessel CAD at 1 and 8 years prior to presentation, respectively. After extensive evaluation and cardiology clearance, including cardiac catheterization showing patent CABGs, informed consent was obtained for right radical nephrectomy and thrombectomy with possible CPB. Tumor thrombus levels were IIIb hepatic and IIId supradiaphragmatic infra-atrial for Patient #1 and Patient #2, respectively (6).

A modified chevron incision was used, commencing approximately two fingerbreadths below the right costal margin, and extending out laterally to the mid-axillary line. The right kidney was mobilized laterally and posteriorly, and the perirenal collateral circulation was ligated. The renal artery was approached using the posterior approach after medial rotation of the renal hilum, then it was ligated and divided (7). The collateral circulation collapsed, making the remaining dissection easier to perform.

**Figure 1:**
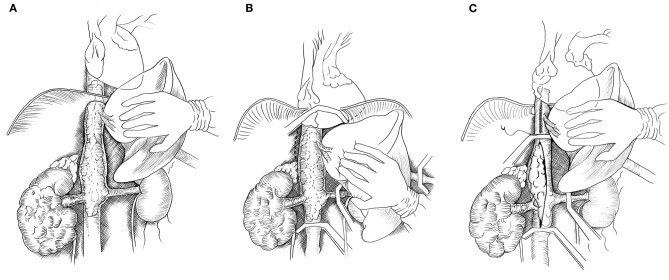
Drawing showing abdominal removal of renal cell carcinoma with level IIId tumor thrombus (TT) (supradiaphragmatic, infraatrial extension). **(A)** The abdominal inferior vena cava (IVC) is exposed by mobilizing the liver off the retrohepatic IVC. **(B)** The central tendon of the diaphragm and IVC are dissected off the posterior abdominal wall (dotted lines). The right atrium, distal IVC, porta hepatis and left renal vein are clamped. **(C)** If the tumor thrombus cannot be milked downward below the major hepatic veins (MHVs), then the tumor thrombus is removed from the intrapericardial IVC to below the MHVs and the upper cava is closed. A vascular clamp is repositioned below the MHVs, and the porta hepatis clamp is released permitting hepatic venous drainage during the removal of the TT and closure or reconstruction of the IVC.

The liver was completely mobilized off the IVC using an organ transplant-based approach, with the only remaining structural attachments being the hepatic veins and porta hepatis (Piggyback liver mobilization) (3). In Patient #1 with Level IIIb hepatic TT, the liver dissection as described was sufficient to allow removal of the TT. For Patient #2, due to extension of the TT above the diaphragm, the central diaphragm tendon was dissected to the supra-diaphragmatic area, and the intra-pericardial IVC was identified. The dissection was circumferential so that the intra-pericardial IVC could be encircled below or above the confluence into the RA. The RA was gently pulled beneath the diaphragm and into the abdomen. If more exposure of the RA was required, the central tendon of the diaphragm could be incised at the midline, allowing the pericardium to be exposed, and a pericardiotomy could be performed. Figure 1 demonstrated the steps of abdominal removal of renal cell carcinoma with level IIId tumor thrombus (TT) (supradiaphragmatic, infraatrial extension).

Use of intra-operative transesophageal echocardiography (TEE) was critical to delineate the cranial extent and mobility of the tumor thrombus during dissection of the retrohepatic IVC, supra-diaphragmatic IVC, and RA. In addition, its use ensured that there was no pulmonary artery emboli or TT extending into the RA. In addition, the intra-operative TEE guided us during application of the partial clamp onto the RA, making sure that the clamp excluded tumor and that the coronary sinus was not obstructed.

Furthermore, a plane was created between the IVC and posterior abdominal wall. Small tributaries can become engorged to look like lumbar vessels, and they need to be identified and ligated. The advantage of creating this plane was to facilitate circumferential control of the IVC. Vascular isolation of the IVC was then achieved inferior and superior to the TT and the left and right renal veins.

For Patient #1 with level IIIb TT, once the liver and IVC were completely mobilized via the Piggyback technique, vascular clamps were placed in the infra-renal vena cava followed by the left renal vein. The TT was then milked below the major hepatic veins, and the IVC was clamped without the need for a Pringle maneuver.

TT of Patient #2 could not be “milked” downward out of the intra-pericardial IVC, as Patient #2's TT was bulky and not freely mobile. In this case, a Pringle maneuver was performed to temporarily occlude blood inflow to the liver for 20 min with no change in liver function tests. Vascular clamps were placed in the infra-renal vena cava, followed by the left renal vein, and a Satinsky clamp was placed across the RA (under TEE monitoring) or across the intrapericardial or suprahepatic IVC. The IVC was incised from the diaphragm to the renal vein, and the TT of Patient #2 was removed. In some areas, the TT was dissected sharply off the IVC wall. The three major hepatic veins were visualized, and their orifices were inspected, leading to a small TT being removed from the right major hepatic vein. Following removal of TT and closure of the upper cava, the vascular clamp was repositioned below the hepatic veins. The Pringle maneuver was discontinued, and blood flow to the liver was re-established. Clamping below the major hepatic veins allows for a time wise short Pringle maneuver. The remaining IVC below the hepatic veins was sutured closed. The TT was removed en-bloc along with the right kidney tumor (Figure 1C).

At the end of performing surgery on each patient, a TEE was re-performed to rule out any pulmonary artery emboli or piece of TT left behind.

Blood loss and packed red blood cell (PRBC) transfusions were 1,000 and 1,200 cc and 3U and 2U of PRBC, respectively. Pathology examination revealed 7 and 5 cm renal cell carcinomas of clear and papillary cell types, respectively; both tumors were Fuhrman grade III. Both tumors were pT3b Nx M0 (8). The right adrenal gland was free of tumor in both patients. Both patients were discharged home on day 7. Patient #1 had subsequently multiple bone metastases and died at 1 year after surgery. Patient #2 remained stable without noticeable disease and was followed for 3 years after surgery. Perioperative characteristics of both patients are summarized in Table 1.

**Table 1:** Perioperative characteristics of Patient #1 and Patient #2.

	**Patient #1**	**Patient #2**
**Preop**		
Age (years)	56	68
Reason for CABG/Timing prior to presentation for renal cell cancer	2 Vessel CAD/1 Year	3 Vessel CAD/8 Years
Clinical diameter (cm)	5	6.5
Tumor thrombus	IIb hepatic	IIId supradiaphragmatic infra-atrial
**Intraop**		
Pringle's Maneuver	No	Yes
Blood transfusion	3U PRBCs	2U PRBCs
Estimated blood loss (cc)	1,000	1,200
**Postop**		
Pathological diameter (cm)	7	5
Pathological staging	pT3b Nx M0	pT3b Nx M0
**Follow up**	Bone metastasis and died at 1-year post-surgery.	No noticeable recurrence or metastasis during the 3-years follow-up period.

*CABG, Coronary artery bypass graft; CAD, Coronary artery disease; PRBCs, Packed red blood cells; U, unit*.

## Discussion

RCC that includes TT requires challenging and complex surgery for its removal usually via a multidisciplinary team approach. Dealing with RCC and TT either below or above the diaphragm forced us to think how to resect them without using CBP (9, 10). The goal was to avoid sternotomy and complications related to CPB (11). Redo sternotomy due to prior CABG is technically challenging, because of increased risk of adhesions and injury to critical structures (5).

There have been reports of two other patients having RCC and the presence of CAD (12, 13), including one RCC case with TT extending into the RA in a patient with previous sternotomy (13), with good outcomes observed in both patients. Our patients were different in that we were able to remove the TT below and above diaphragm without CPB as previously described (9, 10), and our surgeries were also successful.

However, no advanced plans were made in case one or both of our patients had pulmonary emboli or if the TT could not be resected via the abdominal approach. After critical review, we plan to follow a preparatory surgical strategy of placing a venous cannula via the right internal jugular vein into the RA. Once the abdomen is opened, we will place a purse string into the distal IVC and abdominal aorta. The distal IVC will be used as long as it is not obstructed by the TT or bland thrombus (14). If the TT embolizes to the pulmonary artery (PA), the patient will be immediately heparinized, and CPB will be initiated by cannulation of the aorta, IVC, and internal jugular vein. A re-sternotomy will then be performed, dissecting the heart, allowing access to the PA and right atrium. If there is tumor in the RA, then the internal jugular cannula will be removed from the RA and then placed in the superior vena cava (SVC), snaring the SVC. Once the RA is opened, the TT can be removed from the abdomen. The main PA is then opened, and the tumor emboli are removed as previously described (15). If the tumor is confined to the RA, an alternative to performing sternotomy is to open the RA via a small right thoracotomy, which allows for the TT removal. A drawback to this technique is that if the tumor embolizes to the main PA, the use of a sternotomy may become necessary.

Aggressive surgical management of RCC with TT extending into the IVC and RA is currently the only treatment modality able to achieve any long-term survival in these patients (4), and previous CABG should not be a limiting factor in the surgical decision.

Removal of RCC with extension of TT into the retrohepatic IVC and up to the RA continues to be an operative challenge. We described our organ transplant-based approach in dealing with RCC and TT via a trans-abdominal approach using a modified Chevron incision with avoidance of CPB (1, 9, 10). Recently, Gill et al. described a case report of resection of RCC/TT level IV using the robotic surgery assisted with mini-thoracotomy (16). The two patients described here benefited from this surgical approach, but their clinical outcomes could have been different if there was any indication for sternotomy such as sudden onset of uncontrollable bleeding, pulmonary tumor thrombus emboli and/or the requirement to emergently place the patient on CPB. Moreover, large intracardial TT thrombus may be too difficult to be removed without sternotomy (6, 9, 10). Our approach is always to start and remove the TT transabdominally. We will certainly plan to be prepared differently for the next surgery, as CPB may be required, and maximum utilization of available strategies will hopefully help to improve patient outcomes in challenging cases as those described here.

## Data Availability Statement

The original contributions generated for the study are included in the article/supplementary material, further inquiries can be directed to the corresponding author.

## Ethics Statement

The studies involving human participants were reviewed and approved by Institutional Review Board, Miller School of Medicine, University of Miami. The patients/participants provided their written informed consent to participate in this study.

## Author Contributions

GC: concept, design, data collection, data analysis, and interpretation. AF and TS: critical revision of article. All authors drafting and approval of article.

## Conflict of Interest

The authors declare that the research was conducted in the absence of any commercial or financial relationships that could be construed as a potential conflict of interest.

## Abbreviations

RCC: Renal cell carcinoma
IVC: Inferior vena cava
CPB: Cardiopulmonary bypass
TT: Tumor thrombus
CABG: Coronary artery bypass graft
CAD: Coronary artery disease
RA: Right atrium
TEE: Transesophageal echocardiography
PRBC: Packed red blood cell
SVC: Superior vena cava.
